# Optimization of the Electrospun Niobium–Tungsten Oxide Nanofibers Diameter Using Response Surface Methodology

**DOI:** 10.3390/nano11071644

**Published:** 2021-06-23

**Authors:** Babajide Oluwagbenga Fatile, Martin Pugh, Mamoun Medraj

**Affiliations:** Department of Mechanical, Industrial and Aerospace Engineering, Concordia University, Montreal, QC H3G 1M8, Canada; Oluwagbenga.fatile@mail.concordia.ca (B.O.F.); martin.pugh@concordia.ca (M.P.)

**Keywords:** niobium–tungsten oxide, nanofibers, electrospinning, optimization, response surface methodology

## Abstract

The present research aimed to investigate the effect of working parameters on the electrospinning of niobium–tungsten oxide nanofibers and optimize the process using central composite design (CCD) based on the response surface methodology (RSM). An experiment was designed to assess the effects of five variables including the applied voltage (V), spinning distance (D), polymer concentration (P), flow rate (F), and addition of NaCl (N) on the resulting diameter of the nanofibers. Meanwhile, a second-order prediction model of nanofibers diameter was fitted and verified using analysis of variance (ANOVA). The results show that the diameter of the nanofibers was significantly influenced by all the variables except the flow rate. Some second-order and cross factor interactions such as VD, DP, PF, PN, and P^2^ also have significant effects on the diameter of the nanofibers. The results of the ANOVA yielded *R*^2^ and adjusted *R*^2^ values of 0.96 and 0.93 respectively, this affirmed that the predictive model fitted well with the experimental data. Furthermore, the process parameters were optimized using the CCD method and a maximum desirability function of 226 nm was achieved for the diameter of the nanofibers. This is very close to the 233 nm diameter obtained from a confirmatory experiment using the optimum conditions. Therefore, the model is representative of the process, and it could be used for future studies for the reduction of the diameter of electrospun nanofibers.

## 1. Introduction

Over the last few years, researchers have been focusing on the fabrication of ceramic nanofibers with large surface-to-volume ratios, as these materials have potential applications where high porosity is desirable [1]. In the previous decades, there were difficulties in synthesizing one-dimensional nanostructures of high purity due to the lack of suitable manufacturing routes [2]. Various techniques for fabricating nanofibers had been reported in the literature and some of the most widely used techniques include flash spinning, self-assembly, phase separation, drawing-processing, electrospinning, template-assisted synthesis, melt blowing, electrochemical deposition and solvent casting [3,4,5,6]. Among these methods, electrospinning has become the most widely used technique due to its low cost, simplicity, high yield, tunable porosity, high surface-to-volume ratio, control over various process parameters, and ability to control the nanofiber composition [7,8]. Electrospinning is regarded as an efficient method of synthesizing nanofibers due to its ability to process different types of polymers and its consistency in synthesizing nanofibers with controllable morphology and diameter [2]. Additionally, the electrospinning technique is beneficial for fabricating nanostructures from varieties of raw materials. This method combines the use of electrospray and spinning processes to achieve a highly efficient technique suitable for spinning different types of fibers from polymer solutions or melts [2]. These unique advantages of electrospinning have attracted researchers from different fields for synthesizing different nanostructures for various applications, such as optical electronics, healthcare, filtration, biomedical, defense and security, nanocatalysis, environmental engineering, biotechnology, protective clothing, pharmaceutical, and nanofiber reinforced composites [7,9].

Electrospinning has been widely used for fabricating nanofibers from most organic polymers because it is easier to prepare a polymer solution with the required rheological properties for electrospinning [10,11]. This has motivated several researchers to carry out investigations on electrospun polymer nanofibers and the technique has been majorly used for fabricating polymer nanofibers. On the other hand, ceramics are generally considered not to be electrospinnable alone, except at high temperatures when they are in a molten state [12]. Recent efforts [3,13,14] by several researchers have led to the fabrication of ceramic nanofibers through electrospinning using spinnable precursors. Typical procedures for fabricating ceramic nanofibers involve the preparation of an electrospinnable sol by dissolving precursor salt and a polymer in a suitable solvent: the next step is to spin the prepared solution to produce composites nanofibers consisting of the precursor salt and a carrier polymer, while the last step is to sinter the electrospun nanofibers composite at high temperatures to remove the associated organic components [14,15].

Most recently, ceramic nanowires are being explored as potential electrode materials for electrochemical energy storage devices. One of the ceramic nanowires currently being explored for this purpose is niobium–tungsten oxide nanowires [16]. This material has been reported to exhibit open crystal structures as well as valence state changes of niobium and tungsten ions [16]. These unique attributes offer high specific capacity and cycling performance which makes it suitable for storing lithium (Li) ions without any noticeable structural changes [16]. The theoretical capacity of niobium–tungsten oxide nanowires is 293.56 mAh g^−1^ [17], this is significantly higher than those of H_2_Ti_12_O_25_ (229 mAh g^−1^) [18], Li_4_Ti_5_O_12_ (175 mAh g^−1^) [19], and Li_2_Ti_3_O_7_ (198 mAh g^−1^) [20]. Besides, niobium–tungsten oxide nanowires have demonstrated, significant structural stability, high power density, as well as environmental friendliness in comparison with other potential anode materials [17]. Several approaches including sol-gel and solid-state methods have been used to fabricate niobium–tungsten oxide nanowires. However, these methods have been reported to involve complicated processes [16,17,18,19,20,21]. As a result of this, it is desirable to develop a simple, versatile and highly efficient technique for fabricating niobium–tungsten oxide nanowires. In 2017, Yan et al. [16] made the first attempt to fabricate niobium–tungsten oxide nanowires using the electrospinning technique and these authors also studied the lithium-ion storage capability of the material. In 2018, the authors went further by characterizing niobium–tungsten oxide nanowires as a potential anode for lithium-ion batteries (LiBs) [17].

Over the last few decades, various attempts had been made to understand the effects of working parameters such as applied voltage, solution composition, electric field strength, type of collector, and polymer solution flow rate on the diameter and morphology of electrospun ceramic nanofibers such as TiO_2_ [22]. However, the control of nanofibers’ diameter, morphology, and properties still poses some challenges. Moreover, the combination of precursor salt, solvent and polymer usually influence the behavior of the electrospinning solution. Thus, it is imperative to understand how the contents of the electrospinning solution and other working parameters influence the morphology and properties of electrospun ceramic nanofibers, this will help in selecting a combination of suitable processing parameters for fabricating nanofibers with desired characteristics for various applications.

The use of various statistical experimental design methods for studying the effects of variables on chemical processes had been reported by several authors [23,24,25,26]. These tools are useful for the design of experiments, construction of numerical models, evaluation of the effects of variables, and optimization of processes. Among the available experimental design methods, Response Surface Model (RSM) has been widely used by various researchers for process optimization [23,27,28]. RSM utilizes a set of advanced experimental design techniques that makes it easier to study the effects of factors on a system and optimize the response. It is also suitable for fitting a second-order prediction equation for the response from fewer experimental results: the quadratic terms in the model equation are useful for modeling curvature in the true response function and this provides additional information for a better understanding of a process [29]. Central Composite Design (CCD) is one of the most widely used RSM. It is an advanced factorial design with center points, complimented with a star or axial points [29]. The additional centre and star points help to increase the accuracy of the estimate for the first and second-order terms in the model equation [29]. The use of CCD is suitable for this current research. However, it can be practically impossible in certain processes to perform experiments at the extreme levels of some variables which tend to be a drawback of this design [30]. Nevertheless, optimization of the electrospinning process for fabricating nanofibers is currently receiving attention, demonstrating the suitability of this design for the optimization of the electrospinning process [31,32,33]. In addition to RSM, other systematic approaches with new algorithms and designs are currently being explored for materials optimization [34,35,36].

The process of fabricating niobium–tungsten oxide nanofibers and their potential as an anode material for LIBs had been reported [16,17]. To the best of our knowledge, the effects of working parameters on the electrospinning of niobium–tungsten oxide nanofibers have not been investigated. To this end, this current research aims at (1) investigating the effects of applied voltage, spinning distance, polyvinylpyrrolidone (PVP) content, flow rate, and addition of sodium chloride (NaCl) on the morphology and diameter of niobium–tungsten nanofibers, (2) developing a response surface model (Box–Wilson Central Composite Design (CCD)) to predict the diameter of niobium–tungsten nanofibers, and (3) finding the optimum conditions for fabricating niobium–tungsten oxide nanofibers with minimum diameter.

## 2. Materials and Methods

### 2.1. Design of Experiment

The experimental design and results analysis in this research were carried out using JMP Pro version 9.s.2 software (SAS Institute Inc., Cary, NC, USA). Potential parameters that can influence the electrospinning process are numerous and it is difficult to investigate all of them in one single research due to time and cost. In this regard, series of preliminary investigations and extensive literature review were carried out to select the most influential factors. Furthermore, the factors selected in this research are similar to those investigated by other authors [22,27,33].

Before optimization, experiments were conducted using a screening design that generated sixteen experimental runs. The screening experiment was much more useful because it provided the feasibility range for each factor to obtain uniform nanofibers. The results obtained from the screening experiments are summarized in Table 1. After the screening experiments were completed, the Box–Wilson Central Composite Design (CCD) was utilized to design experiments involving five continuous factors. The factors included applied voltage, spinning distance, polymer concentration, flow rate, and addition of NaCl at five coded levels, + α, +1, 0, −1, and −α (the value of α is 2.00 in this work) as shown in Table 2.

The CCD generated thirty-six experimental runs including ten as the replication of the central points. All the experiments were executed in random order and the corresponding values of the diameter of the nanofibers were recorded as the response.

### 2.2. Preparation of the Precursor Solution

The precursor solution for electrospinning was prepared using niobium oxalate (98%, Sigma Aldrich, Oakville, ON, Canada), ammonium metatungstate hydrate (99.5%, Sigma Aldrich, Oakville, ON, Canada), polyvinylpyrrolidone (MW 1,300,000 gmol^−1^, 100% purity, Sigma Aldrich, Oakville, ON, Canada), ethanol (96.9%, Sigma Aldrich, Oakville, ON, Canada) and nitric acid (99.5%, Fischer Scientific, Ottawa, ON, Canada). All the materials used in this research were of analytical grade. Firstly, a certain amount of NaCl (as listed in Table 3) was dissolved in the mixture of deionized water (5 mL) and ethanol (15 mL) with continuous stirring for 12 h. This was followed by the addition of 0.5 g niobium oxalate, 0.2 g ammonium metatungstate, and 1.0 g citric acid to the mixture with continuous stirring for 24 h to obtain a homogeneous solution. Then, a variable amount of PVP was added according to the experimental design in Table 3. The resulting mixture was stirred continuously for 12 h to obtain a viscous precursor solution. The procedure was repeated for preparing the precursor solution for all the experimental runs.

### 2.3. Electrospinning of Nanofibers

**Figure 1:**
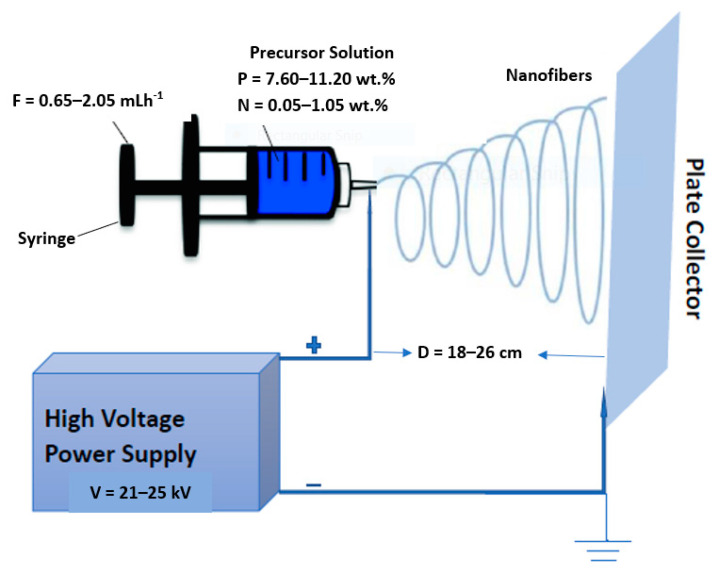
Schematic diagram of the electrospinning process.

The niobium–tungsten oxide nanofibers were fabricated based on the experimental design in Table 2. All experiments were randomly carried out to eliminate systematic bias in the responses. The fabrication process was carried out by transferring the precursor solution into a plastic syringe with a stainless steel needle. An aluminum foil was employed as the collector with the spinning distance of 18–26 cm, while the flow rate of the pump was varied between 0.65 and 2.05 mL h^−1^ A potential difference of (21–25 kV) was applied to the droplet of the precursor solution at the tip of the needle, the application of high voltage results in the deformation of the droplet. The polymer jet is ejected into the electric field, and this causes the jet to undergo a bending movement under columbic repulsion and the polymer jet is stretched into nanofibers before being deposited on the collector. A schematic diagram of the electrospinning process is shown in Figure 1.

### 2.4. Measurement of the Nanofibers Diameter

**Figure 2:**
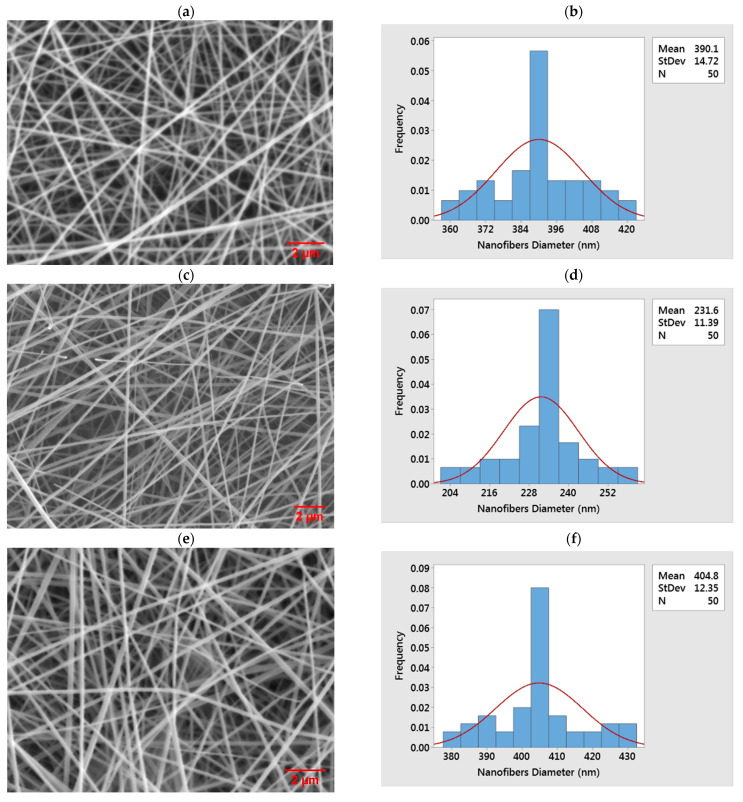

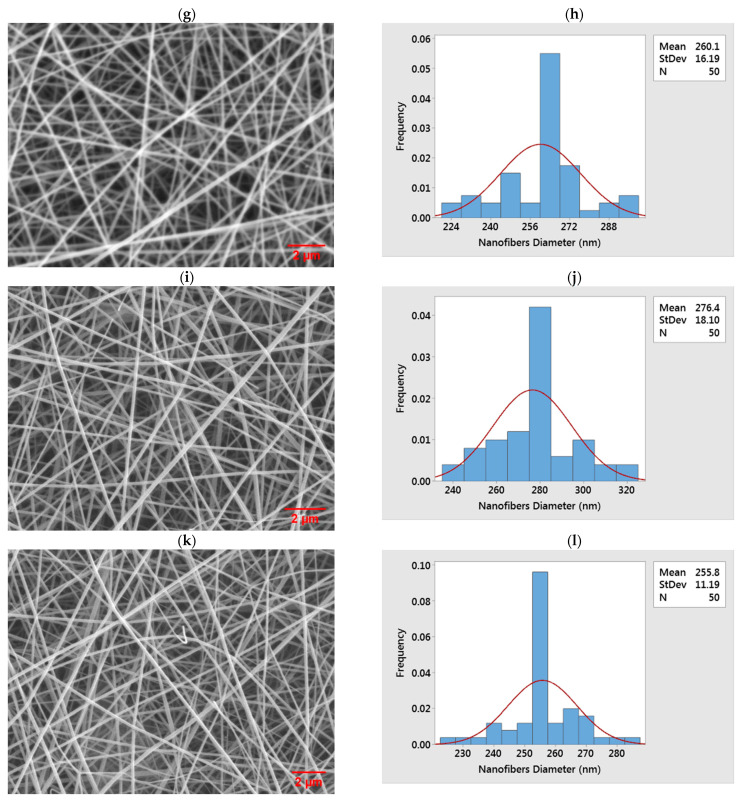
SEM micrographs and nanofibers diameter distribution corresponding to the experimental run numbers in Table 3, as follows: (**a**,**b**) 3, (**c**,**d**) 13, (**e**,**f**) 14, (**g**,**h**) 18, (**i**,**j**) 29, and (**k**,**l**) 36.

Micrographs of the nanofibers fabricated under different experimental conditions were obtained using a Scanning Electron Microscope (SEM) (HITACHI, Chiyoda-Ku, Tokyo, Japan). The average nanofiber diameter for each of the experiments was determined by measuring the diameter of 50 randomly selected nanofibers (Figure 2) using Image J software [37]. Before the average nanofibers diameter for each of the experiments was determined, the diameter of the selected nanofibers was initially measured at various spots, and it was observed that each of the nanofibers has a fairly uniform diameter. The results obtained for each of the experiments are presented in Table 3.

### 2.5. Development of the CCD Response Surface Model

A model Equation (1) that describes the diameter of the niobium–tungsten oxide nanofibers (*df*) as a function of the five factors (*V*, *D*, *P*, *F*, and *N*) was determined in terms of the coded factors by performing multiple regression analysis on the experimental data.

An analysis of variance (ANOVA) of the response was also conducted to evaluate the full second-order polynomial approximation of the response surface model. The significance of each coefficient of the model equation was determined using the corresponding *p*-value.

## 3. Results and Discussions

### 3.1. Estimation of Coefficients in the Mathematical Model Equation

The result of the multiple regression analysis (Table 4) yielded Equation (1) which represents a mathematical relationship between the response (nanofibers diameter, *df*) and the factors in a coded unit:

(1) $$\begin{array}{c} df=2887-112.1\;V-49.3\;D-127\;P-252\;F+463\;N+13.42\;P^{2}-16.1\;F^{2}\\ -39.5\;N^{2}+4.07\;VD+9.09\;VF-14.43\;VN-5.01\;DP-5.74\;DF\\ +9.54\;DN+25.06\;PF-28.40\;PN-42.80\;FN \end{array}$$

The significance of each parameter in the model equation including quadratic, cross-factor interactions, and linear was evaluated to affirm the effect of each term in the model together with their interactions using ANOVA at 95% confidence level and probability values (*p*-values) from Fisher’s (*F*) exact test. At 95% confidence level, model equation parameters with *p*-values less than 0.05 are significant while the model equation parameters with *p*-values greater than 0.05 are non-significant [24,28,38]. The results obtained from this study show that the terms *V*, *D*, *P*, *N*, *VD*, *DP*, *PF*, *PN*, and *P*^2^ were significant in the model. The *f*-value was also used to confirm the level of significance of the model terms. The level of significance was based on the magnitude of *f*-values, with a higher value representing a larger influence on the process being studied [23]. Hence, the results obtained from the *f*-values are in agreement with those of the *p*-values.

### 3.2. Verification of the Response Surface Model

**Figure 3:**
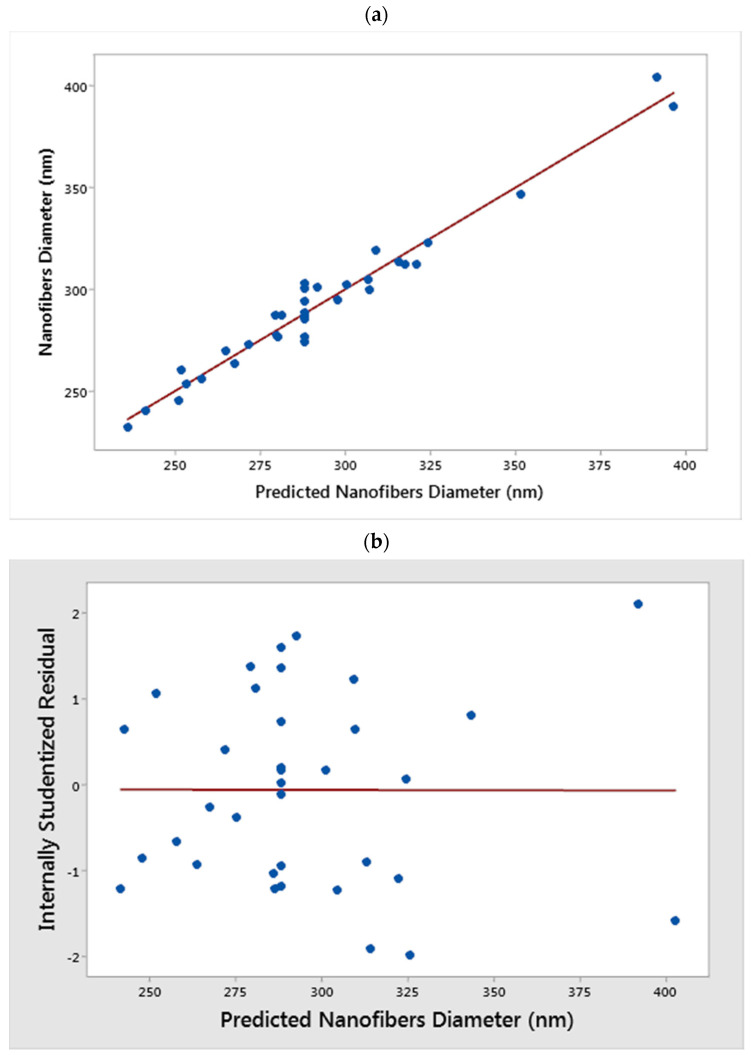
Validation of the nanofibers diameter model using (**a**) observed experimental data versus predicted values (**b**) internally studentized residuals versus predicted value.

The efficiency of the developed model was verified by computing the linear correlation coefficient as shown in Figure 3a. This was used to obtain the determination coefficient (*R*^2^) and the adjusted *R*^2^ for the model by plotting the diameter of the experimental nanofibers against the model predicted nanofibers diameter. The value of the (*R*^2^ = 0.96) shows that only 4.0% of the total variations are not explained by the model. Additionally, the value obtained for the adjusted *R*^2^ (0.93) is high, *R*^2^ and adjusted *R*^2^ close to 1.0 indicate that there are minor discrepancies between the predicted and experimental nanofibers diameter [23,24,28]. The assumption of the constant variance was also confirmed using the plot of the internally studentized residual against the predicted values. The results presented in Figure 3b show that the sample points were randomly scattered within the outlier detection limits of −2 to +2 [28]. This confirms the correlation of the prediction model with the experimental data.

The accuracy of the prediction model was also verified by ANOVA. The results presented in Table 4 show that the regression model is highly significant owing to its very low *p*-value. The insignificant lack of fit also affirms that the predictive model fitted well with the observed data [23,27,28]. All these results show that the predicted model is accurate, and it is reliable for representing and optimizing the diameter of the nanofibers.

### 3.3. Visualization of the Interactions Between the Model Parameters

**Figure 4:**
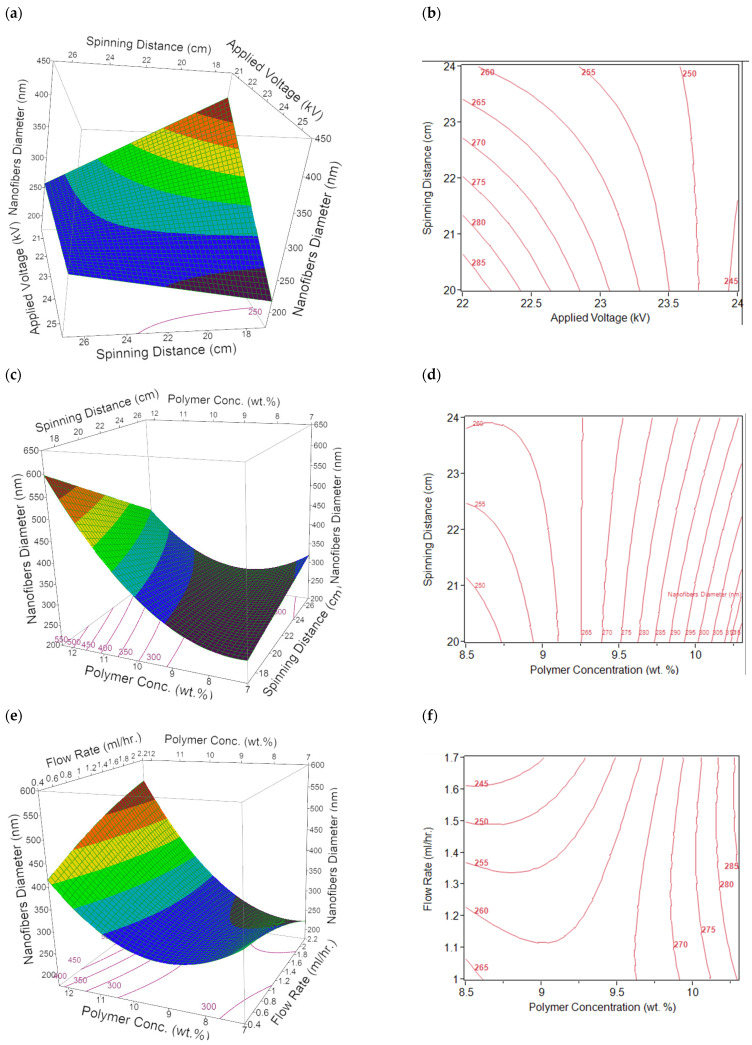

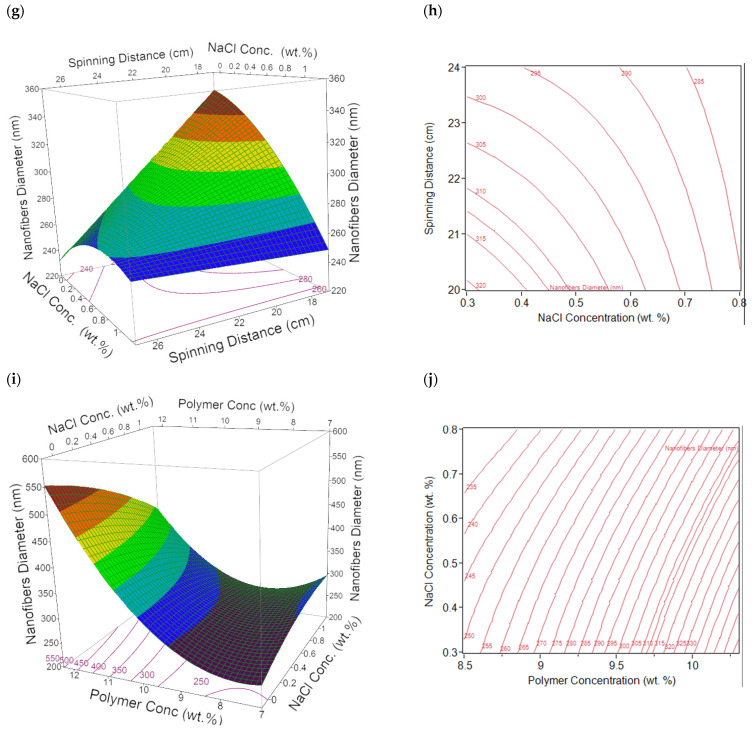
3D response surface plots (**a**,**c**,**e**,**g**,**i**) and 2D contour plots (**b**,**d**,**f**,**h**,**j**) of interaction between the (**a**,**b**) applied voltage and spinning distance (*VD*); (**c**,**d**) spinning distance and polymer concentration (*DP*); (**e**,**f**) flow rate and polymer concentration (*PF*); (**g**,**h**) spinning distance and concentration of NaCl (*DN*); and (**i**,**j**) polymer concentration and concentration of NaCl (*PN*).

The three-dimensional (3D) response surfaces and two-dimensional (2D) contour plots of the cross-factor interaction effects between the model parameters are presented in Figure 4. The surface of the model parameters and interaction between two variables were presented while other parameters were kept constant. The cross-factor interaction effect between the applied voltage and spinning distance is shown in Figure 4a,b. It is observed that the nanofibers diameter is highly dependent on both applied voltage and spinning distance as diameter generally decreases with increasing applied voltage and spinning distance. However, an applied voltage above 23 kV has a larger influence than the spinning distance. The decrease in nanofibers diameter with increasing applied voltage and spinning distance could be attributable to the fact that an increase in the applied voltage enhances the electrostatic force on the solution, this usually causes the polymer jet to be stretched further thereby leading to the formation of thinner nanofibers [22,39]. Furthermore, evaporation of solvent usually occurs after the polymer jet has been stretched into nanofibers and before deposition on the collector. Thus, the optimum spinning distance is required for complete evaporation of the solvent before the nanofibers reach the collector [7]. This will also result in more stretching of the nanofibers thereby reducing the diameter [7,38]. Therefore, the combined effect of applied voltage and spinning distance at the optimum level results in the reduction of the diameter of the nanofibers.

Figure 4c,d depict the interaction between spinning distance and polymer concentration. It is observed that the cross-factor interaction between the predictors significantly affects the diameter of the nanofibers. The nanofiber diameter increases with increasing polymer concentration at low spinning distance. At high spinning distance, the diameter of the nanofibers initially decreases with increasing polymer concentration up to 9 wt %. Nonetheless, the nanofiber diameter increases with increasing spinning distance beyond 9 wt % polymer concentration. This trend could be attributable to the fact that the optimum distance required to stretch the nanofibers was attained at 9 wt % polymer concentration. Therefore, an increase in the spinning distance could not stretch the nanofibers further. Thus, the effect of polymer concentration becomes the dominant factor. This trend is in line with the results obtained by other authors [22,40,41]. Generally, a minimum solution concentration is required for the formation of nanofibers during the electrospinning process. The results of the investigation carried out by previous authors revealed that a mixture of beads and discontinuous nanofibers were obtained at low polymer concentration, and as the solution concentration increases, smooth and uniform nanofibers with increased diameter were obtained [40,42].

Figure 4e,f show the cross-factor interaction between the polymer concentration and the flow. These figures show that the diameter of the nanofibers decreases with increasing flow rate at low polymer concentration. However, the effect of flow rate on the diameter of the nanofibers was reversed at high polymer concentration as the nanofiber diameter increases with increasing flow rate. As the flow rate increases at high polymer concentration, the influence of polymer concentration becomes dominant, and this causes an increase in the fiber diameter. Moreover, Figure 4g,h reveal the cross-factor interaction between spinning distance and concentration of NaCl. These figures show that the diameter of the nanofibers decreases with increasing spinning distance at low NaCl concentration. Contrarily, the effect was opposite at high NaCl concentration as the nanofibers’ diameter slightly decreases with increasing spinning distance. The combined effect of spinning distance and NaCl concentration at the optimum level results in a 24% reduction in nanofiber diameter. Finally, Figure 4i,j show the interaction between polymer concentration and concentration of NaCl. The figures reveal that interaction between the two variables has a significant effect on the diameter of the nanofibers. This diameter generally decreases with increasing NaCl concentration at high polymer concentration. However, the effect was inverted at low polymer concentration as the nanofiber diameter slightly increases with increasing NaCl concentration. Beachley and Wen [43] explained that the addition of salts to the polymer solution increases the conductivity and the surface charge density of the solution jet thereby resulting in the formation of beadless nanofibers with reduced diameter. In the current research, a 28% reduction in the nanofiber diameter was obtained by changing the NaCl concentration from 0.05 to 1.05 wt %. Furthermore, the addition of NaCl to the precursor solution also prevented the formation of beaded nanofibers. This trend is consistent with the results reported by other authors [41,43].

### 3.4. Optimization and Validation of the Response Surface Model

The optimum experimental conditions of the five variables; the applied voltage (*V*), spinning distance (*D*), polymer concentration (*P*), flow rate (*F*), and NaCl concentration (*N*) have been determined to meet the previously set goal of minimizing the diameter of the nanofibers. The maximum desirability function was achieved with nanofiber diameter of 226 nm under optimum conditions of 24 kV applied voltage, 20 cm spinning distance, 8.5 wt % polymer concentration, 1.7 mL h^−1^, flow rate, and 0.8 wt % NaCl concentration. The model has been validated by conducting another experiment using the obtained optimum conditions. The experimental result obtained under the optimum conditions is 233 nm, which is very close to the 226 nm predicted value. This further confirms the reliability of the developed model. It also implies that this method can be successfully employed for fabricating niobium–tungsten oxide nanofibers with controlled morphology and diameter for various applications.

**Figure 5:**
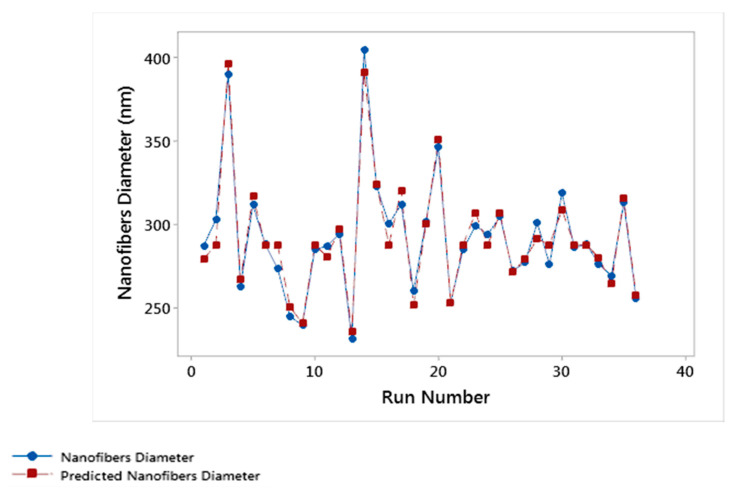
Model predicted nanofibers diameter in relation to the current experimental results.

Furthermore, the model was verified using the graphical representation of the experimental and predicted values of the diameter of the nanofibers shown in Figure 5. The values were also used to calculate the average model accuracy (*AMA*) using Equation (2) [33,44] where *X*_i_ is the experimental nanofibers diameter at the run (i), *Y*_i_ is the predicted nanofibers diameter at the run (i), and *n* is the total number of experimental runs.

(2) $$AMA=\sum_{i=I}^{n}\frac{\;\left[1-ABS\;\frac{\left(X_{i}-Y_{i\;}\right)}{X_{i}}\right]\;}{n}\times 100$$

The result obtained from Equation (2) shows an average model accuracy of 98%, this is considered as an acceptable model [37,38].

## 4. Conclusions

The influence of process parameters on the electrospinning of niobium–tungsten oxide nanofibers was investigated and optimized using the response surface method. The predictive model developed using RSM together with CCD was found to be accurate and reliable for representing the diameter of the nanofibers. The reliability of the predictive model was assessed using ANOVA and linear correlation coefficient. The determination coefficient (*R*^2^) and the adjusted *R*^2^ of 0.96 and 0.93 were obtained, with an average model accuracy of 98%. Furthermore, the result of the ANOVA test performed on the model indicates that the applied voltage (*V*)_,_ spinning distance (*D*)_,_ polymer concentration (*P*)_,_ NaCl Concentration (*N*)_,_ and other cross factor interactions such as *VD*, *DP*, *PF*, *PN*, and *P*^2^ have a significant effect on the diameter of the nanofibers. The CCD method was utilized to optimize the process parameters. Under the optimum conditions of the applied voltage (24 kV), spinning distance (20 cm), polymer concentration (8.5 wt %), flow rate (1.7 mL h^−1^), and NaCl concentration (0.8 wt %), experimental nanofibers of 233 nm were obtained. This is very close to the 226 nm diameter predicted by the model. Therefore, the proposed model is representative of the process, and it could be employed as a base for future studies for the reduction of nanofiber diameter within the range of the factors used in the research. Furthermore, the approach presented in this study can be employed as a basis for fabricating uniform ceramic nanofibers for various applications. Nevertheless, it should be noted that the electrospinning of ceramic nanofibers is dependent on the choice of precursor salt and type of polymer. In terms of future work, it is suggested that the influence of other relevant working parameters on the electrospinning of niobium–tungsten oxide nanofibers are investigated using other approaches such as machine learning and artificial neural networks.

## Figures and Tables

**Table 1:** Summary of the preliminary investigation results.

Parameters	Effect on the Nanofibers Diameter and Morphology
Applied Voltage	Electrospinning process requires an applied voltage beyond the critical value before nanofibers can be obtained. During the screening process, nanofibers were not produced when the voltage was below 14 kV. Nonetheless, nanofibers with irregular shapes and sizes were obtained when the voltage was between 15 kV and 18 kV. Further increase in the applied voltage between 19 kV and 27 kV resulted in the formation of uniform nanofibers. As the voltage increased beyond 28 kV, nanofibers with beads and irregular morphology were obtained.
Polymer Conc.	Nanofibers were not obtained when the polymer concentration was below 7.3 wt %. Above a polymer concentration of 7.3 wt %, nanofibers with fairly uniform morphology were obtained. As the polymer concentration exceeded 11.8 wt %, the morphology of the resulting nanofibers became irregular.
Spinning Distance	Nanofibers with large diameters were obtained at a spinning distance below 16 cm, while beaded nanofibers were obtained above a spinning distance of 27 cm.
Flow Rate	Deposition of unspun droplets on the collector was observed when the flow rate was set above 2.2 mL h^−1^. It is also observed that a flow rate below 0.65 mL h^−1^. was not suitable for obtaining continuous nanofibers.
Type of Collector	The screening experiments were carried out using a stationary plate and rotating drum collectors. The results showed that there is no significant difference in the diameter of the nanofibers obtained from the two collectors.

**Table 2:** The factors and associated levels for experimental design.

Factors	Coded Factors	Coded Levels				
		+α	+1	0	−1	−α
Applied Voltage (kV)	V	25	24	23	22	21
Spinning Distance (cm)	D	26	24	22	20	18
Polymer Conc. (wt %)	P	11.20	10.30	9.40	8.50	7.60
Flow Rate (mL hr^−1^)	F	2.05	1.70	1.35	1	0.65
NaCl Conc. (wt %)	N	1.05	0.80	0.55	0.30	0.05

**Table 3:** Experimental design showing randomized list of the niobium–tungsten oxide nanofibers electrospinning experiments and the resulting fibers diameter.

Run Order	Applied Voltage (kV)	Spinning Distance (cm)	Polymer Conc. (wt %)	Flow Rate (mL h^−1^)	Conc. of NaCl (wt %)	Nanofibers Diameter (nm)
1	23	22	9.40	2.05	0.55	287.2
2	23	22	9.40	1.35	0.55	303
3	22	20	10.30	1.70	0.30	390.1
4	23	26	9.40	1.35	0.55	263.1
5	24	24	10.30	1.70	0.30	312
6	23	22	9.40	1.35	0.55	288.2
7	23	22	9.40	1.35	0.55	274
8	24	20	8.50	1.70	0.30	245.2
9	24	24	8.50	1.70	0.80	239.9
10	23	22	9.40	1.35	0.55	285.1
11	23	22	9.40	0.65	0.55	287.3
12	22	20	8.50	1.00	0.30	294.5
13	24	20	8.50	1.00	0.80	231.6
14	23	22	11.20	1.35	0.55	404.8
15	21	22	9.40	1.35	0.55	323.2
16	23	22	9.40	1.35	0.55	300.6
17	24	20	10.30	1.00	0.30	312.3
18	25	22	9.40	1.35	0.55	260.1
19	22	24	8.50	1.00	0.80	302.1
20	22	20	10.30	1.00	0.80	346.5
21	22	24	8.50	1.70	0.30	253.4
22	23	22	9.40	1.35	0.55	285.3
23	24	20	10.30	1.70	0.80	299.4
24	23	22	9.40	1.35	0.55	294.3
25	22	24	10.30	1.70	0.80	305
26	23	22	7.60	1.35	0.55	272.5
27	22	20	8.50	1.70	0.80	277.3
28	23	22	9.40	1.35	0.05	301.1
29	23	22	9.40	1.35	0.55	276.4
30	23	18	9.40	1.35	0.55	319
31	23	22	9.40	1.35	0.55	286.4
32	23	22	9.40	1.35	0.55	288.2
33	24	24	10.30	1.00	0.80	276.4
34	23	22	9.40	1.35	1.05	269.5
35	22	24	10.30	1.00	0.30	313.2
36	24	24	8.50	1.00	0.30	255.8

**Table 4:** Summary of the statistical analysis of the model coefficients and the corresponding *p*-values.

Source	Sum of Squares	DF	*f*-Values	*p*-Values
Model	42,538.1	17	26.94	<0.0001
V	7909.77	1	85.16	<0.0001
D	2622.95	1	28.24	<0.0001
P	21,582	1	232.36	<0.0001
F	4.42	1	0.05	0.8298
N	1086.76	1	11.7	0.0030
VD	1061.13	1	11.42	0.0033
DP	1301.41	1	14.01	0.0015
VF	161.93	1	1.74	0.2033
DF	258.41	1	2.78	0.1126
PF	996.98	1	10.73	0.0042
VN	208.08	1	2.24	0.1518
DN	363.86	1	3.92	0.0633
PN	654.08	1	7.04	0.0162
FN	224.25	1	2.41	0.1376
P2	3782.33	1	40.72	<0.0001
F2	125.22	1	1.35	0.2608
N2	194.54	1	2.09	0.1650
Lack of Fit	899.74	9	1.17	0.4117
Pure Error	772.13	9		
Cor Total	44,209.96	35		

*R*^2^ = 0.96, Adj. *R*^2^ = 0.93.

## Data Availability

Data are contained within the article.

## References

[1] Weber J., Singhai R., Zekri S., Kumar A. (2008). One-dimensional nanostructures: Fabrication, characterization and applications. Int. Mater. Rev..

[2] Wei Q., Xiong F., Tan S., Huang L., Lan E.H., Dunn B., Mai L. (2017). Porous one-dimensional nanomaterials: Design, fabrication and applications in electrochemical energy storage. Adv. Mater..

[3] Barakat N.A.M., Kim B., Kim H.Y. (2009). Production of smooth and pure nickel metal nanofibers by the electrospinning technique: Nanofibers possess splendid magnetic properties. J. Phys. Chem. C.

[4] Lin C., Lai M.O., Lu L., Zhou H., Xin Y. (2013). Structure and high rate performance of Ni^2+^ doped Li _4_Ti_5_O_12_ for lithium-ion battery. J. Power Sources.

[5] Lim S.K., Lee S., Hwang S., Kim H. (2006). Photocatalytic deposition of silver nanoparticles onto organic/inorganic composite nanofibers. Macromol. Mater. Eng..

[6] Ye W., Yu H., Cheng X., Zhu H., Zheng R., Liu T., Long N., Shui M., Shu J. (2018). Highly efficient lithium container based on non-Wadsley-Roth structure Nb_18_W_16_O_93_ nanowires for electrochemical energy storage. Electrochim. Acta.

[7] Bhardwaj N., Kundu S.C. (2010). Electrospinning: A fascinating fiber fabrication technique. Biotechnol. Adv..

[8] Haider A., Haider S., Kang I.K. (2018). A comprehensive review summarizing the effect of electrospinning parameters and potential applications of nanofibers in biomedical and biotechnology. Arab. J. Chem..

[9] Hohman M.M., Shin M., Rutledge G., Brenner M.P. (2001). Electrospinning and electrically forced jets. I. Stability theory. Phys. Fluids.

[10] Subbiah T., Bhat G.S., Tock R.W., Parameswaran S., Ramkumar S.S. (2005). Electrospinning of nanofibers. J. Appl. Polym. Sci..

[11] Li D., Xia Y. (2004). Electrospinning of nanofibers: Reinventing the wheel?. Adv. Mater..

[12] Fridrikh S.V., Yu J.H., Brenner M.P., Rutledge G.C. (2003). Controlling the fiber diameter during electrospinning. Phys. Rev. Lett..

[13] Sarkar J., Khan G.G., Basumallick A. (2007). Nanowires: Properties, applications and synthesis via porous anodic aluminum oxide template. Bull. Mater. Sci..

[14] Li D., McCann J.T., Xia Y., Marquez M. (2006). Electrospinning: A simple and versatile technique for producing ceramic nanofibers and nanotubes. J. Am. Ceram. Soc..

[15] Wu H., Pan W., Lin D., Li H. (2012). Electrospinning of ceramic nanofibers: Fabrication, assembly and applications. J. Adv. Ceram..

[16] Yan L., Lan H., Yu H., Qian S., Cheng X., Long N., Zhang R., Shui M., Shu J. (2017). Electrospun WNb_12_O_33_ nanowires: Superior lithium storage capability and their working mechanism. J. Mater. Chem. A.

[17] Yana L., Shua J., Lib C., Chenga X., Zhua H., Yua H., Zhangb C., Zhengc Y., Xied Y., Guo Z. (2019). W_3_Nb_14_O_44_ nanowires: Ultrastable lithium storage anode materials for advanced rechargeable batteries. Energy Storage Mater..

[18] Akimoto J., Kataokaa K., Kojimaa N., Hayashia S., Gotoha Y., Sotokawab T., Kumashiro Y. (2013). A novel soft-chemical synthetic route using Na_2_Ti_6_O_13_ as a starting compound and electrochemical properties of H_2_Ti_12_O_25_. J. Power Sources.

[19] Zhao Y., Pang S., Zhang C., Zhang Q., Gu L., Zhou X., Li G., Cui G. (2013). Nitridated mesoporous Li_4_Ti_5_O_12_ spheres for high-rate lithium-ion batteries anode material. J. Solid State Electrochem..

[20] Villevieillea C., Thournoutb M.V., Scoyerb J., Tessierc C., Olivier-Fourcadea J., Jumasa J.-C., Monconduit L. (2010). Carbon modified Li_2_Ti_3_O_7_ ramsdellite electrode for Li-ion batteries. Electrochim. Acta.

[21] Saritha D., Pralong V., Varadaraju U.V., Raveau B. (2010). Electrochemical Li insertion studies on WNb_12_O_33_-A shear ReO_3_ type structure. J. Solid State Chem..

[22] Someswararao M.V., Dubey R.S., Subbarao P.S.V., Singh S. (2018). Electrospinning process parameters dependent investigation of TiO_2_ nanofibers. Results Phys..

[23] Li L., Ma Q., Wang S., Song S., Li B., Guo R., Cheng X., Cheng Q. (2018). Photocatalytic performance and degradation mechanism of aspirin by TiO_2_ through response surface methodology. Catalysts.

[24] Bustillo-Lecompte C.F., Mehrvar M. (2016). Treatment of an actual slaughterhouse wastewater by integration of biological and advanced oxidation processes: Modeling, optimization, and cost-effectiveness analysis. J. Environ. Manag..

[25] Rabbi A., Nasouri K., Bahrambeygi H., Shoushtari A.M., Babaei M.R. (2012). RSM and ANN approaches for modeling and optimizing of electrospun polyurethane nanofibers morphology. Fibers Polym..

[26] Homayoni H., Ravandi S.A.H., Valizadeh M. (2009). Electrospinning of chitosan nanofibers: Processing optimization. Carbohydr. Polym..

[27] Sarlak N., Nejad M.A.F., Shakhesi S., Shabani K. (2012). Effects of electrospinning parameters on titanium dioxide nanofibers diameter and morphology: An investigation by Box-Wilson central composite design (CCD). Chem. Eng. J..

[28] Lin Y.P., Mehrvar M. (2018). Photocatalytic treatment of an actual confectionery wastewater using Ag/TiO_2_/Fe_2_O_3_: Optimization of photocatalytic reactions using surface response methodology. Catalysts.

[29] Del Vecchio R.J. (1997). Understanding Design of Experiments: A Primer for Technologists.

[30] Ferreira S.L., Bruns R.E., da Silva E.G., dos Santos W.N., Quintella C.M., David J.M., de Andrade J.B., Breitkreitz M.C., Jardim I.C., Neto B.B. (2007). Statistical designs and response surface techniques for the optimization of chromatographic systems. J. Chromatogr. A.

[31] Topuz F., Holtzl T., Szekely G. (2021). Scavenging organic micropollutants from water with nanofibrous hypercrosslinked cyclodextrin membranes derived from green resources. Chem. Eng. J..

[32] Topuz F., Abdulhamid M.A., Holtzl T., Szekely G. (2021). Nanofiber engineering of microporous polyimides through electrospinning: Influence of electrospinning parameters and salt addition. Mater. Des..

[33] Utkarsh U., Hegab H., Tariq M., Syed N.A., Rizvi G., Pop-Iliev R. (2020). Towards analysis and optimization of electrospun PVP (polyvinylpyrrolidone) nanofibers. Adv. Polym. Technol..

[34] Abdelhakim H.E., Coupe A., Tuleu C., Edirisinghe M., Craig D.Q.M. (2019). Electrospinning optimization of eudragit E PO with and without chlorpheniramine maleate using a design of experiment approach. Mol. Pharm..

[35] Cao B., Adutwum L.A., Oliynyk A.O., Luber E.J., Olsen B.C., Mar A., Buriak J.M. (2018). How to optimize materials and devices via design of experiments and machine learning: Demonstration using organic photovoltaics. ACS Nano.

[36] Hardian R., Liang Z., Zhang X., Szekely G. (2020). Artificial intelligence: The silver bullet for sustainable materials development. Green Chem..

[37] Abramoff M.D., Magalhaes P.J., Ram S.J. (2004). Image processing with ImageJ. Biophotonics Int..

[38] Ray S., Lalman J.A. (2011). Using the Box-Benkhen design (BBD) to minimize the diameter of electrospun titanium dioxide nanofibers. Chem. Eng. J..

[39] Khalil A., Hashaikeh R., Jouiad M. (2014). Synthesis and morphology analysis of electrospun copper nanowires. J. Mater. Sci..

[40] Deitzel J.M., Kleinmeyer J., Harris D., Tan N.C.B. (2001). The effect of processing variables on the morphology of electrospun nanofibers and textiles. Polymer.

[41] Matabola K.P., Moutloali R.M. (2013). The influence of electrospinning parameters on the morphology and diameter of poly (vinylidene fluoride) nanofibers-effect of sodium chloride. J. Mater. Sci..

[42] Haghi A.K., Akbari M. (2007). Trends in electrospinning of natural nanofibers. Phys. Status Solidi..

[43] Beachley V., Wen X. (2009). Effect of electrospinning parameters on the nanofiber diameter and length. Mater. Sci. Eng. C.

[44] Elkasaby M., Hegab H.A., Mohany A., Rizvi G.M. (2018). Modeling and optimization of electrospinning of polyvinyl alcohol. Adv. Polym. Technol..

